# PI3Kα targeting, nipping pancreatic cancer evolution in the bud

**DOI:** 10.15252/emmm.202114362

**Published:** 2021-05-31

**Authors:** Glancis Luzeena Raja Arul, Martin E Fernandez‐Zapico

**Affiliations:** ^1^ Schulze Center for Novel Therapeutics Mayo Clinic Rochester MN USA; ^2^ Department of Molecular Pharmacology and Experimental Therapeutics Mayo Clinic Graduate School of Biomedical Sciences Rochester MN USA

**Keywords:** Cancer, Signal Transduction

## Abstract

Thibault *et al* (2021) elucidate key signalling events mediating metastatic evolution in pancreatic ductal adenocarcinoma (PDAC) by demonstrating a role of PI3Kα in the regulation of macro‐metastatic disease and a corresponding pro‐tumoural immune response supporting disease progression.

## Graphical abstract

M. Fernandez‐Zapico and G. Raja Arul discuss the study by Thibault *et al* (in this issue of EMBO Molecular Medicine) that demonstrates a role of PI3Kα in the regulation of macro‐metastatic pancreatic ductal adenocarcinoma (PDAC) and disease progression.

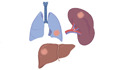

PDAC is one of the most commonly occurring pancreatic neoplasms, accounting for 95% of all cancers derived from this organ. Despite intensive research in systemic and individualized therapeutics over the last four decades, the 5‐year survival rate for PDAC has only seen a modest increase from 3 to 10% (Cancer.org, 2021). Failure of current therapeutic approaches to control metastatic PDAC, or prolong life, necessitates further research into genetic/epigenetic drivers and identification of suitable targets to prevent rapid progression to metastatic, chemoresistant disease. The search for a distinct gene signature to distinguish localized vs. metastatic PDAC by profiling cell‐free DNA (cfDNA) led Thibault and colleagues to define a new role of the PI3K (phosphatidylinositol‐3‐kinase) pathway in PDAC biology. PI3K signalling mediates functions such as cell growth, motility and proliferation, hyperactivation of which results in tumour development and progression, making it a master regulator of cancer (Yang *et al,* 2019). Inhibition of PI3K signalling could therefore be a significant therapeutic target and has proven effective in the treatment of several cancers (Alagesan *et al,* 2015). While previous preclinical studies and clinical trials have linked PI3K signalling with tumour progression, the implications of this pathway on the aggressiveness of disease and regulation of immune elements remained unaddressed.

Thibault *et al* (2021) aimed to identify key signalling events driving the evolution of metastasis in PDAC. In this study, they highlight the association of a higher PI3Kα activation signature in primary PDAC with a worse prognosis, making it a strong prognostic marker for aggressive disease. PI3Kα is the p110α catalytic subunit of PI3K. Mutations in the PI3Kα gene have been reported to increase the PI3K kinase activity in many cancers of the breast, brain, liver and lungs. Using two preclinical models to validate the role PI3Kα plays in PDAC evolution, they showed that PI3Kα activity mediated progression from micro‐metastatic to macro‐metastatic disease. The authors use cfDNA in their study as a serum biomarker to evaluate disease aggressiveness. CfDNA, derived from plasma during liquid biopsies, is extensively used in clinical oncology as a biomarker for early cancer detection and disease progression (Corcoran & Chabner, 2018). Several studies over the past decade have explored the use of cfDNA for applications beyond cancer screening, such as predicting␣disease prognosis, and monitoring the efficacy of administered chemotherapy in patients (Schwarzenbach *et al,* 2011). In this study, the authors use cfDNA to monitor the efficacy of pharmacologic PI3Kα inhibition in halting the evolution of metastatic disease in PDAC. They further demonstrate a corresponding immune response, where PI3Kα activity increases pro‐tumoural characteristics in immune cells by increasing IL‐3 (interleukin 3) production. This triggers an increase in TNFα (tumour necrosis factor alpha) production by macrophages, further promoting tumour cell migration.

The authors further validated the association of a PI3K activation signature with poor prognosis in two independent cohorts of PDAC patients. They next used pharmacological inhibition of PI3K (using pan‐PI3K and isoform‐specific inhibitors) to demonstrate concentration‐dependent inhibition of cell motility and directed cell migration in both human and murine cancer cells. Concomitant inhibition of FGFR and PI3K via drug treatment did not significantly alter cell migration compared to individual inhibition, implying that the PI3Kα pro‐migratory signal was in part due to FGFR activation. They further demonstrated that PI3Kα inhibition halted cell migration and decreased cell survival irrespective of the PDAC cells genetic landscape (including genetic alterations on *KRAS, PIK3CA* and *PTEN*). This finding is of particular significance in PDAC as genetic/epigenetic heterogeneity is often the underlying cause of treatment failure in clinical settings (Lomberk *et al,* 2018; Peng *et al,* 2019). The ability of PI3K inhibitors to impact PDAC cells regardless of their genetic/epigenetic landscape make PI3K an ideal pharmacological target for PDAC patients, and further makes the case for the prognostic potential of PI3Kα activity signature in the detection of micro‐metastatic disease.

The authors further established a correlation between cfDNA levels and PI3K activity in patient tumours, highlighting the efficacy of using cfDNA to establish PI3K activity as a biomarker for micro‐metastatic disease. Comparison of resected tumours from patients, with and without pathological nodal involvement, revealed high pAKT substrate levels and thus an increase in PI3K/AKT activity in these tumours. These patients also exhibited higher levels of cfDNA with mutated KRAS, which could potentially reflect on a dependency to PI3K activity. The KPC mouse model is a well‐established model for PDAC that exhibits distinct characteristics often observed in human PDAC (Lee *et al,* 2016). The authors showed that longitudinal average levels of cfDNA positively correlated with disease progression and lethality in this model. Further, they identified a distinct 160–210 bp fragment of cfDNA that was more present in mice with metastatic PDAC than in mice with localized PDAC. This finding is of particular significance in clinical settings as it would bolster the prediction rate for patient survival. Murine pancreatic cancer cells injected into nude mice were used to generate a second preclinical model, which confirmed that PI3K inhibition halted the progression of micro‐metastatic foci. Consistent with the results obtained in the KPC model, pharmacologic inhibition of PI3K in xenografts prevented metastatic growth in the lungs.

The immune system plays a multi‐faceted role in PDAC progression. While PDAC is generally characterized by immune suppression as a result of tumour microenvironment regulation by cancer cells, some immune cells promote metastasis (Inman *et al,* 2014). The authors therefore turned their attention to the effects of PI3K inhibition on immune response. Pharmacological inactivation of PI3Kα reduced IL‐3 levels in mutant KRAS PDAC cells. PDAC patients with high PI3Kα activity also exhibited a significant increase of the γδ T lymphocytes gene signature, which is associated with differential macrophage differentiation. In their KPC model, the authors showed that PI3Kα inhibition decreased macrophage differentiation into tumour‐associated inflammatory macrophages and reduced TNF‐α production. Further assessment of macrophage function revealed that exogenous TNF‐α increased cancer cell migration and PI3Kα inhibition impaired TNF‐α‐mediated migration (Fig 1).

**Figure 1:**
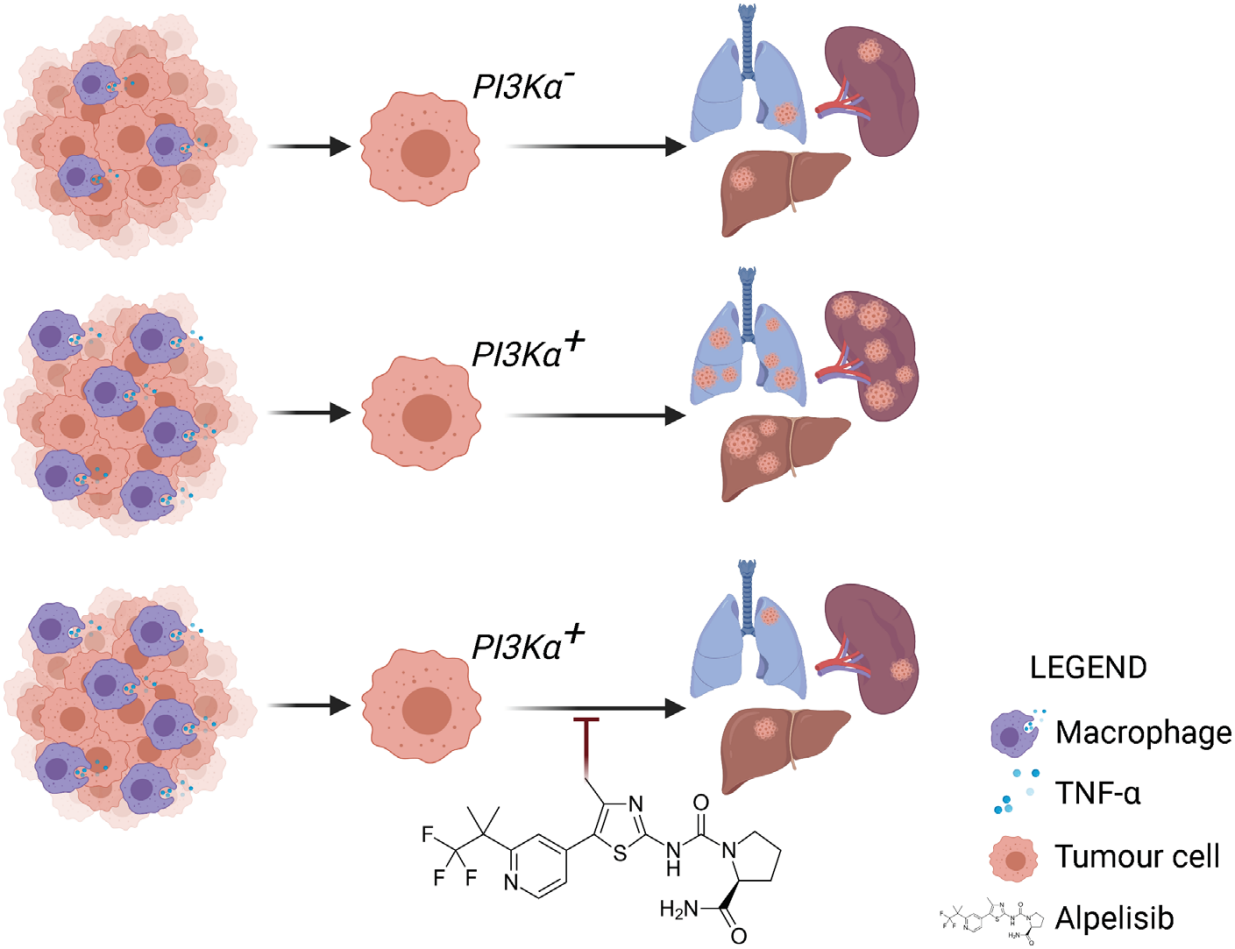
PI3Kα activity drives macro‐metastatic disease evolution Pharmacological inhibition of PI3Kα was used to establish its role in the regulation of macro‐metastatic evolution in pancreatic ductal adenocarcinoma. A positive PI3Kα activation signature in tumour cells mediated by increased TNF‐α secretion from macrophages induced metastatic dissemination to the lungs, liver and spleen. Inhibition of PI3Kα with Alpelisib reduced TNF‐α secretion and metastasis in distant organs. (Image generated on biorender.com, licence from Department of Biochemistry and Molecular Biology, Mayo Clinic Graduate School of Biomedical Sciences, USA).

The results of this study vary from other studies in the field as the authors chose to specifically target PI3Kα, as opposed to using pan‐PI3K inhibitors to investigate their hypothesis. This prevents the recurrent issue of compensatory signals towards the MAPK pathway, which reduces the efficacy of PI3K inhibitors in clinical settings. Another key finding is that a minimal PI3Kα activity is sufficient to promote migration and metastasis. Furthermore, the efficacy of PI3Kα inhibition is not affected by the genetic heterogeneity often observed in PDAC patients.

In conclusion, Thibault *et al* uncovered the prometastatic action of PI3Kα via FGFR‐induced tumour migration and increased TNF‐α secretion by macrophages. PI3Kα emerges as a key regulator in the evolution of macro‐metastatic disease in PDAC. Their findings establish PI3Kα as a suitable pharmacological candidate to prevent the activation of a migratory phenotype in PDAC patients.
